# An extensive analysis of the prognostic and immune role of FOXO1 in various types of cancer

**DOI:** 10.1590/1414-431X2024e13378

**Published:** 2024-05-03

**Authors:** Jie Li, Chao Wang, Xiao Xu, Jun Chen, Haijun Guo

**Affiliations:** 1 Department of Hepatobiliary and Pancreatic Surgery, Affiliated Hangzhou First People's Hospital, West Lake University School of Medicine, Hangzhou, China; 2 Zhejiang University School of Medicine, Hangzhou, China; 3 Key Laboratory of Integrated Oncology and Intelligent Medicine of Zhejiang Province, Hangzhou, China

**Keywords:** FOXO1, Prognostic analysis, Immune analysis, Pan-cancer

## Abstract

Forkhead Box O1 (FOXO1) has been reported to play important roles in many tumors. However, FOXO1 has not been studied in pan-cancer. The purpose of this study was to reveal the roles of FOXO1 in pan-cancer (33 cancers in this study). Through multiple public platforms, a pan-cancer analysis of FOXO1 was conducted to obtained FOXO1 expression profiles in various tumors to explore the relationship between FOXO1 expression and prognosis of these tumors and to disclose the potential mechanism of FOXO1 in these tumors. FOXO1 was associated with the prognosis of multiple tumors, especially LGG (low grade glioma), OV (ovarian carcinoma), and KIRC (kidney renal clear cell carcinoma). FOXO1 might play the role of an oncogenic gene in LGG and OV, while playing the role of a cancer suppressor gene in KIRC. FOXO1 expression had a significant correlation with the infiltration of some immune cells in LGG, OV, and KIRC. By combining FOXO1 expression and immune cell infiltration, we found that FOXO1 might influence the overall survival of LGG through the infiltration of myeloid dendritic cells or CD4+ T cells. Functional enrichment analysis and gene set enrichment analysis showed that FOXO1 might play roles in tumors through immunoregulatory interactions between a lymphoid and a non-lymphoid cell, TGF-beta signaling pathway, and transcriptional misregulation in cancer. FOXO1 was associated with the prognosis of multiple tumors, especially LGG, OV, and KIRC. In these tumors, FOXO1 might play its role via the regulation of the immune microenvironment.

## Introduction

The global incidence and death rates of cancer are increasing at an alarming rate (1). Cancer is the primary reason for mortality and a major obstacle to improving life span in nations globally (2). Finding new biomarkers to diagnose and treat cancers is urgent.

FOXO1, the first gene discovered in the FoxO family, functions as a transcriptional controller implicated in growth, programmed cell death, metabolic processes, and the reaction to stress (3). FOXO1 has gained significant interest in recent times as a promising molecular target for inhibiting cancer. According to reports, FOXO1 has significant involvement in various types of cancer, such as hepatocellular cancer, pancreatic cancer, gastric cancer, and others (4-9).

However, the functions and processes of FOXO1 in different types of cancers remain incompletely understood. By analyzing extensive epigenomic, genomic, proteomic, and transcriptome data from various tumors published on multiple public platforms, a pan-cancer study can detect shared characteristics and variations in specific molecules across different types of cancer (10). Pan-cancer analysis plays a crucial role in tumor diagnosis and treatment by artificially identifying the manifestation and altering characteristics of molecules across various tumors (11). The aim of this study was to acquire FOXO1 expression patterns in diverse tumors, investigate the correlation between FOXO1 expression and tumor prognosis, and uncover the potential mechanisms of FOXO1 in various malignancies.

## Material and Methods

### Data sources

The study included a total of 33 tumors, which are referred to as pan-cancer collectively (Table 1). Data on gene expression profiles and clinical information for pan-cancer were acquired from the Cancer Genome Atlas (TCGA) database, accessible at https://portal.gdc.cancer.gov/. We acquired immunohistochemical (IHC) images of healthy and cancerous human tissues from the Human Protein Atlas database (HPA, https://www.proteinatlas.org/). Moreover, the associations between FOXO1 expression and immune cells (such as B cells, CD4+ T cells, CD8+ T cells, myeloid dendritic cells, and macrophages) were examined using the Tumor Immune Estimation Resource 2.0 (TIMER2.0, http://timer.cistrome.org/), a website with the original data from TCGA database. The STRING database (https://string-db.org/) provided a protein-protein interaction (PPI) network consisting of 100 genes associated with FOXO1.

**Table 1:** Types of cancer evaluated in this study and their abbreviations.

Abbreviations	Type of cancer
ACC	adrenocortical carcinoma
BLCA	bladder urothelial carcinoma
BRCA	breast invasive carcinoma
CESC	cervical and endocervical cancers
CHOL	cholangiocarcinoma
COAD	colon adenocarcinoma
DLBC	diffuse large B-cell lymphoma
ESCA (including ESAD and ESCC)	esophageal carcinoma
ESAD	esophageal adenocarcinoma
ESCC	esophageal squamous cell carcinoma
GBM	glioblastoma multiforme
HNSC	head and neck squamous cell carcinoma
KICH	kidney chromophobe
KIRC	kidney renal clear cell carcinoma
KIRP	kidney renal papillary cell carcinoma
LAML	acute myeloid leukemia
LGG	brain lower grade glioma
LIHC	liver hepatocellular carcinoma
LUAD	lung adenocarcinoma
LUSC	lung squamous cell carcinoma
MESO	mesothelioma
OV	ovarian serous cystadenocarcinoma
PAAD	pancreatic adenocarcinoma
PCPG	pheochromocytoma and paraganglioma
PRAD	prostate adenocarcinoma
READ	rectum adenocarcinoma
SARC	sarcoma
SKCM	skin cutaneous melanoma
STAD	stomach adenocarcinoma
TGCT	testicular germ cell tumors
THCA	thyroid carcinoma
THYM	thymoma
UCEC	uterine corpus endometrial carcinoma
UCS	uterine carcinosarcoma
UVM	uveal melanoma

### Prognosis analysis

In pan-cancer, the relationship between FOXO1 expression and prognosis was evaluated via Kaplan-Meier analysis with log-rank test, based on the TCGA database. Overall survival (OS), disease-specific survival (DSS), and progression-free interval (PFI) were included in the prognosis analysis.

### Relationship between clinical traits through correlation analysis

Through the above prognostic analysis, the prognosis of some tumors was found to be significantly related with FOXO1 expression. Subsequently, an analysis was conducted to examine the correlation between the expression of FOXO1 and the clinical features of these tumors. Correlation analysis involved the utilization of the Wilcoxon rank-sum test and the Spearman rank test.

### Establishment and evaluation of the nomogram

Through the above prognostic analysis, the OS of some tumors was found to be significantly related with FOXO1 expression. Nomogram models were established using tumors from the TAGA database that had a sample size exceeding 500. Afterwards, calibration curves were used to test the accuracy of the nomograms in predicting outcomes for one, three, and five years. Statistical analysis was performed using the Kaplan-Meier method and log-rank test.

### Immune infiltration analysis

Through the above prognostic analysis, the OS of some tumors was found to be significantly related with FOXO1 expression. These tumors were selected for immune infiltration analysis using TIMER2.0. Additionally, the impact of immune cell infiltration on OS was separately examined after categorizing FOXO1 in these tumors.

### PPI network analysis, functional enrichment analysis, and gene set enrichment analysis

The PPI network related to FOXO1 was obtained from the STRING database and 100 genes were included. The interaction threshold was 0.4. An analysis of gene ontology (GO) was conducted using the aforementioned 100 genes. GO encompasses biological pathways (BP), cellular components (CC), and molecular functions (MF). Kyoto Encyclopedia of Genes and Genomes (KEGG) analysis was also performed. Gene set enrichment analysis (GSEA) was then performed using the DESeq R package and the clusterProfiler R package.

First, data preparation involved downloading gene expression data and corresponding annotation files for three types of cancers - kidney renal clear cell carcinoma (KIRC), low grade glioma (LGG), and ovarian carcinoma (OV) - from the TCGA database. We used DESeq2 version 1.8.1 and edgeR version 3.10.2 to normalize paired mRNA sequencing data and to analyze the differentially expressed genes (DEGs) between high- and low-FOXO1 expression groups with |log2[fold change (FC)]| >1 and adjusted P value <0.05 in each cancer type. A Wald test was used in DESeq2 for statistical analysis and calculating P values for the significance of differentially expressed mRNAs in each cancer type. For both tools, we designed the model matrix with high- and low-FOXO1 expression groups. Consistent format functional gene set files were prepared for subsequent Gene Set Enrichment Analysis (GSEA) analysis. The results of this analysis were then used as input in the clusterProfiler R package with the following parameters: nPerm=1000, minGSSize=10, maxGSSize=1000, and P-value-Cutoff=0.05. Correlation analyses of FOXO1 with all genes was performed using TCGA data. Pearson's correlation coefficients were calculated.

### Statistical analysis

Statistical analysis was performed using R (version 4.0.2). It was determined that a P-value less than 0.05 was statistically significant.

## Results

### FOXO1 expression in various types of cancer

**Figure 1:**
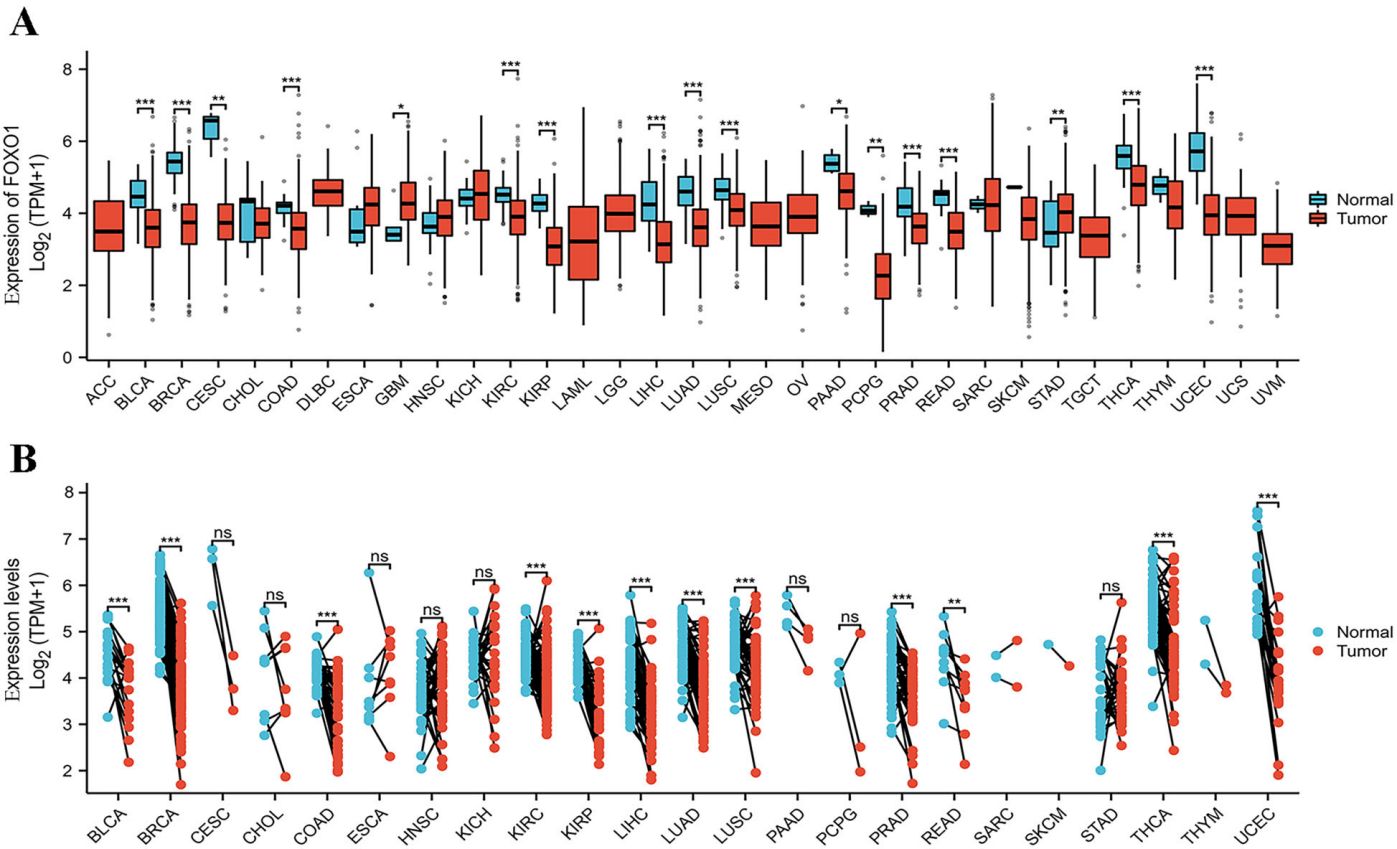
mRNA expression of FOXO1 in pan-cancer. **A**, mRNA expression of FOXO1 in pan-cancer with un-paired samples from Cancer Genome Atlas (TCGA) database. **B**, mRNA expression of FOXO1 in pan-cancer with paired samples from TCGA database. See Table 1 for abbreviations. *P<0.05, **P<0.01, ***P<0.001 (*t*-test). ns: not significant.

According to the TCGA database, the mRNA levels of FOXO1 were found to be significantly higher in STAD and GBM compared to the normal tissues. The mRNA levels of FOXO1 were significantly reduced in BLCA, BRCA, CESC, COAD, KIRC, KIRP, LIHC, LUAD, LUSC, PAAD, PCPG, PRAD, READ, THCA, and UCEC (Figure 1A) (see Table 1 for abbreviations). Additionally, the analysis included the examination of FOXO1 expression in 23 different types of tumors with paired samples from TCGA. Figure 1B displays a significant reduction in FOXO1 mRNA expression across BLCA, BRCA, COAD, KIRC, KIRP, LIHC, LUAD, LUSC, PRAD, READ, THCA, and UCEC.

**Figure 2:**
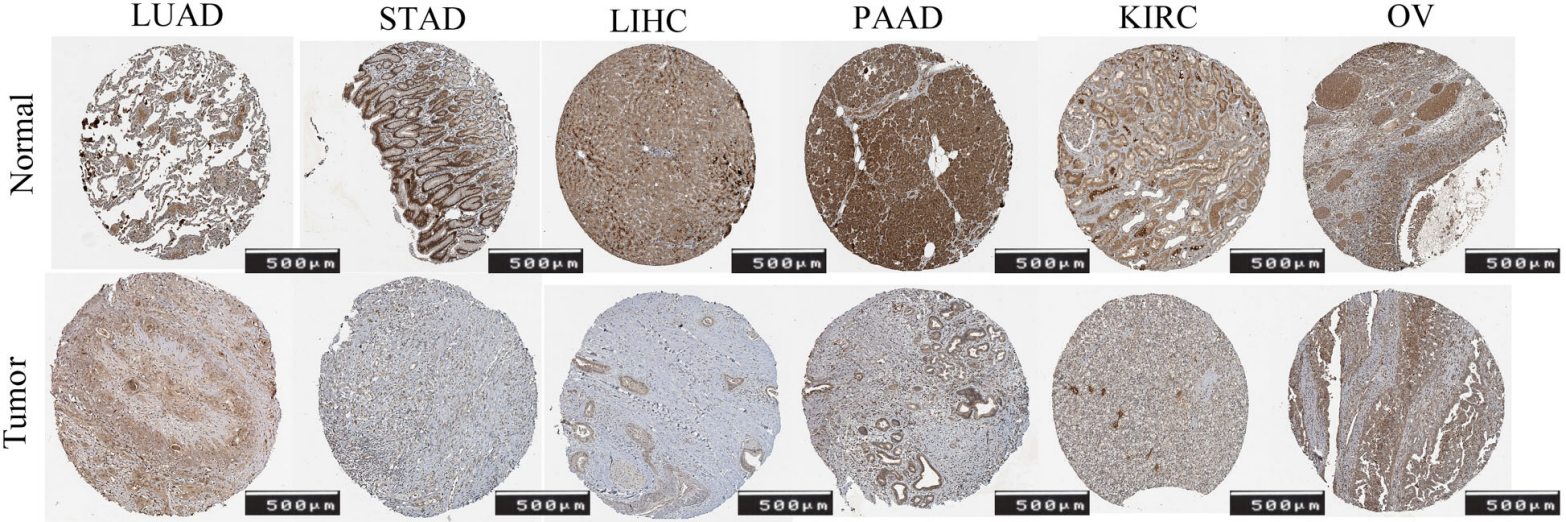
Immunohistochemical images of FOXO1 in human normal and cancerous tissues from the Human Protein Atlas (HPA) database. Scale bar 500 μm. See Table 1 for abbreviations.

Figure 2 displays the IHC images of FOXO1 in LUAD, STAD, LIHC, PAAD, KIRC, and OV, both in human normal and tumor tissue.

### Pan-cancer prognostic analysis of FOXO1

**Figure 3:**
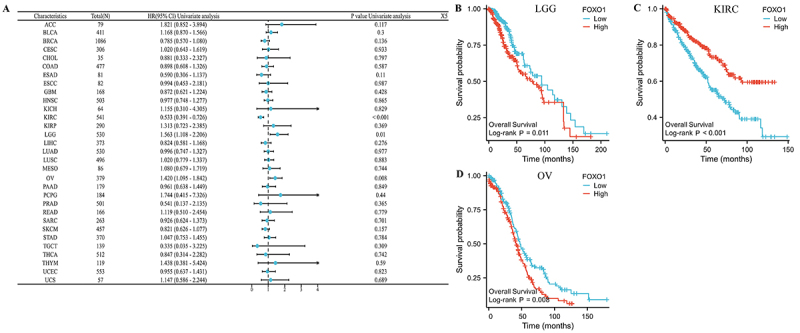
The relationship between FOXO1 expression and overall survival (OS) in several cancers. See Table 1 for abbreviations. **A**, The effect of FOXO1 expression on OS in pan-cancer is shown by the forest map. **B**-**D**, FOXO1 expression was significantly related to the OS of LGG, KIRC, and OV.

The relationship between prognosis of pan-cancer and FOXO1 expression was evaluated using Kaplan-Meier analysis, as per the TCGA database. As a first step, we examined the correlation between FOXO1 expression and OS in various forms of cancer (Figure 3A). The findings indicated a significant correlation between the expression of FOXO1 and OS in LGG, KIRC, and OV. In LGG and OV, a higher level of FOXO1 expression was associated with a worse OS, whereas in KIRC, it was linked to a better OS (Figure 3B-D).

**Figure 4:**
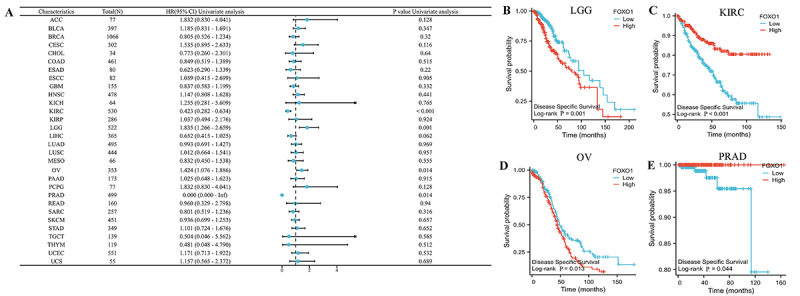
The relationship between FOXO1 expression and disease-specific survival (DSS) in pan-cancer. See Table 1 for abbreviations. **A**, The effect of FOXO1 expression on DSS in pan-carcer was showed by the forest map. **B**-**E**, FOXO1 expression was significantly related to the DSS of LGG, KIRC, OV, and PRAD, respectively.

We then examined the correlation between the expression of FOXO1 and DSS in various types of cancer (Figure 4A). In LGG, KIRC, OV, and PRAD, there was a significant correlation between FOXO1 expression and DSS. In LGG and OV, a higher level of FOXO1 was associated with worse DSS, whereas in KIRC and PRAD, a higher level was linked to improved DSS (Figure 4B-E).

**Figure 5:**
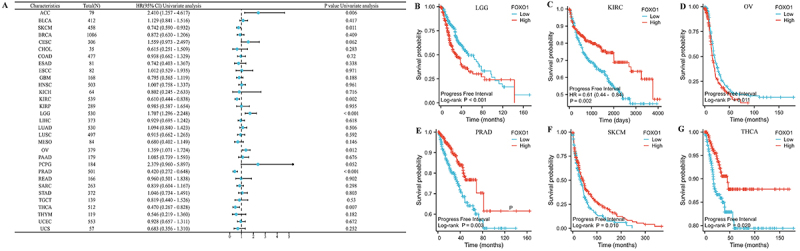
The relationship between FOXO1 expression and progression-free interval (PFI) in pan-cancer. See Table 1 for abbreviations. **A**, The effect of FOXO1 expression on PFI in pan-cancer is shown by the forest map. **B**-**G**, FOXO1 expression was significantly related to the PFI of LGG, KIRC, OV, PRAD, SKCM, and THCA, respectively.

Finally, we examined the correlation between the expression of FOXO1 and PFI across various types of cancer (Figure 5A). There was a significant correlation between FOXO1 expression and PFI in LGG, KIRC, OV, PRAD, SKCM, and THCA. Increased FOXO1 expression was associated with worse PFI in LGG and OV, but correlated with improved PFI in KIRC, PRAD, SKCM, and THCA (Figure 5B-G).

### Correlation between FOXO1 expression and various clinical characteristics

**Figure 6:**
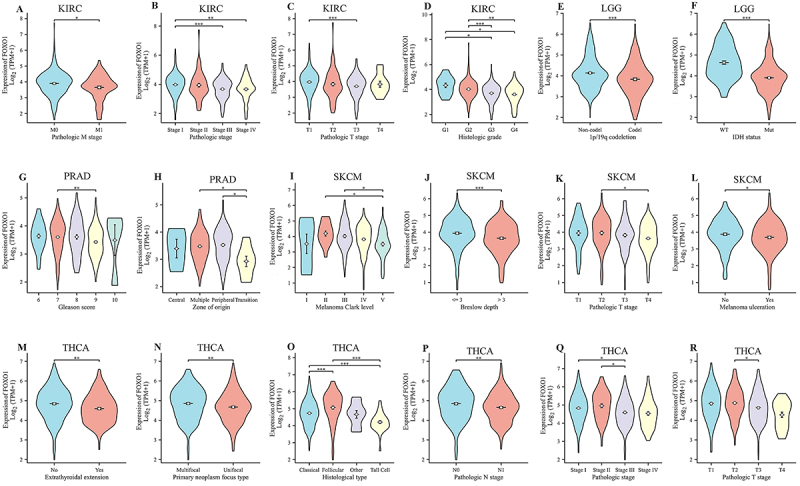
The relationship between FOXO1 expression and clinical characteristics in pan-cancer. See Table 1 for abbreviations. Clinical characteristics that related to FOXO1 expression are shown in panels **A**-**D**, KIRC; **E** and **F**, LGG; **G** and **H**, PARD; **I**-**L**, SKCM; and **M**-**R**, THCA.

The expression of FOXO1 was found to be related to 6 tumors, namely LGG, KIRC, OV, PRAD, SKCM, and THCA, according to the prognostic analysis. Following this, we examined the correlation between the expression of FOXO1 and the clinical characteristics of these tumors. In KIRC, the findings indicated a correlation between FOXO1 expression and pathologic M stage, pathologic stage, pathologic T stage, and histologic grade (Figure 6A-D). The correlation between FOXO1 expression and 1p/19q co-deletion and IDH status in LGG was observed (Figure 6E and F). In PRAD, the correlation between FOXO1 expression and Gleason score and zone of origin was observed (Figure 6G and H). In SKCM, correlations between FOXO1 expression and melanoma Clark level, Breslow depth, pathologic T stage, and melanoma ulceration were observed (Figure 6I-L). In THCA, correlations between FOXO1 expression and extrathyroidal extension, primary neoplasm focus type, histological type, pathologic N stage, pathologic stage, and pathologic T stage were observed (Figure 6M-R).

### Creating and assessing the nomogram models in LGG, KIRC, and OV

**Figure 7:**
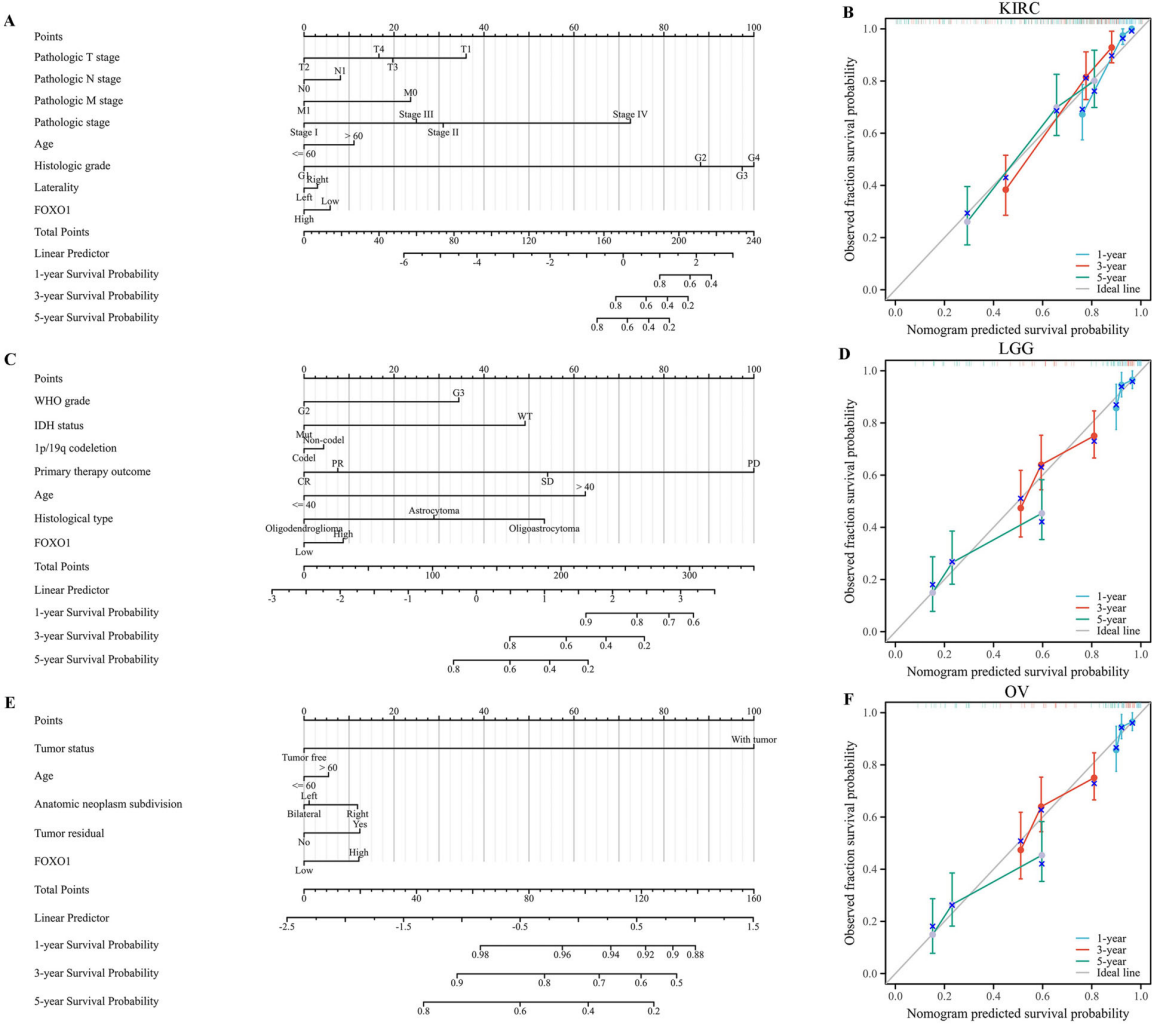
Establishment and evaluation of the nomogram in kidney renal clear cell carcinoma (KIRC), low grade glioma (LGG) and ovarian carcinoma (OV). **A**, A nomogram model in KIRC. **B**, Calibration curve was conducted to evaluate the prediction accuracy of the nomograms model in KIRC at 1, 3, and 5 years. **C**, A nomogram model in LGG. **D**, Calibration curve was conducted to evaluate the prediction accuracy of the nomograms model in LGG at 1, 3, and 5 years. **E**, A nomogram model in OV. **F**, Calibration curve was conducted to evaluate the prediction accuracy of the nomograms model in OV at 1, 3, and 5 years.

The expression of FOXO1 was found to be associated with OS in LGG, KIRC, and OV, as per the prognostic analysis. In the TCGA database, the sample size of LGG, KIRC, and OV exceeded 500 each. Then, nomogram models were established for these tumors. The findings indicated that FOXO1 had a notable impact on the prognosis and demonstrated a strong predictive capacity for OS in KIRC, KIRC, and OV (as depicted in Figure 7A, C, and E). In addition, the nomogram model exhibited a strong accuracy in predicting OS of KIRC, as evidenced by the well-calibrated 1-year, 3-year, and 5-year survival prediction curves shown in Figure 7B. Similar results were found for LGG (Figure 7D) and OV (Figure 7F).

### FOXO1 expression and immune infiltration analysis

**Figure 8:**
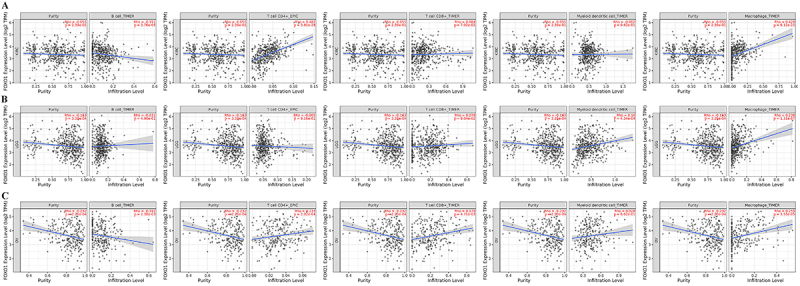
Correlation between FOXO1 expression and immune cell infiltration. Correlation between five immune cell infiltration scores (B cell, T cell CD4+, T cell CD8+, myeloid dendritic cell, and macrophage) and FOXO1 expression in (**A**) kidney renal clear cell carcinoma (KIRC), (**B**) low grade glioma (LGG), and ovarian carcinoma (OV) (**C**).

Immune cells are widely recognized as having a significant impact on the immune microenvironment and could impact the prognosis of cancer patients. However, it is not clear whether FOXO1 could affect the recruitment of immune cells. To explore the possible impact of FOXO1 on prognosis, LGG, KIRC, and OV were examined for correlations between immune cell infiltration and FOXO1 expression. Based on the TCGA database, calculations were performed to determine the scores of five different types of immune cells, namely B cell, CD4+ T cell, CD8+ T cell, myeloid dendritic cell, and macrophage. Figure 8A shows a strong association between FOXO1 expression and the presence of B cells, CD4+ T cells, and macrophages in KIRC. Figure 8B shows a strong association between FOXO1 expression and the presence of myeloid dendritic cells and macrophages in LGG. In the context of OV, the expression of FOXO1 showed a notable association with the infiltration of B cells, CD4+ T cells, CD8+ T cells, and macrophages, as depicted in Figure 8C.

**Figure 9:**
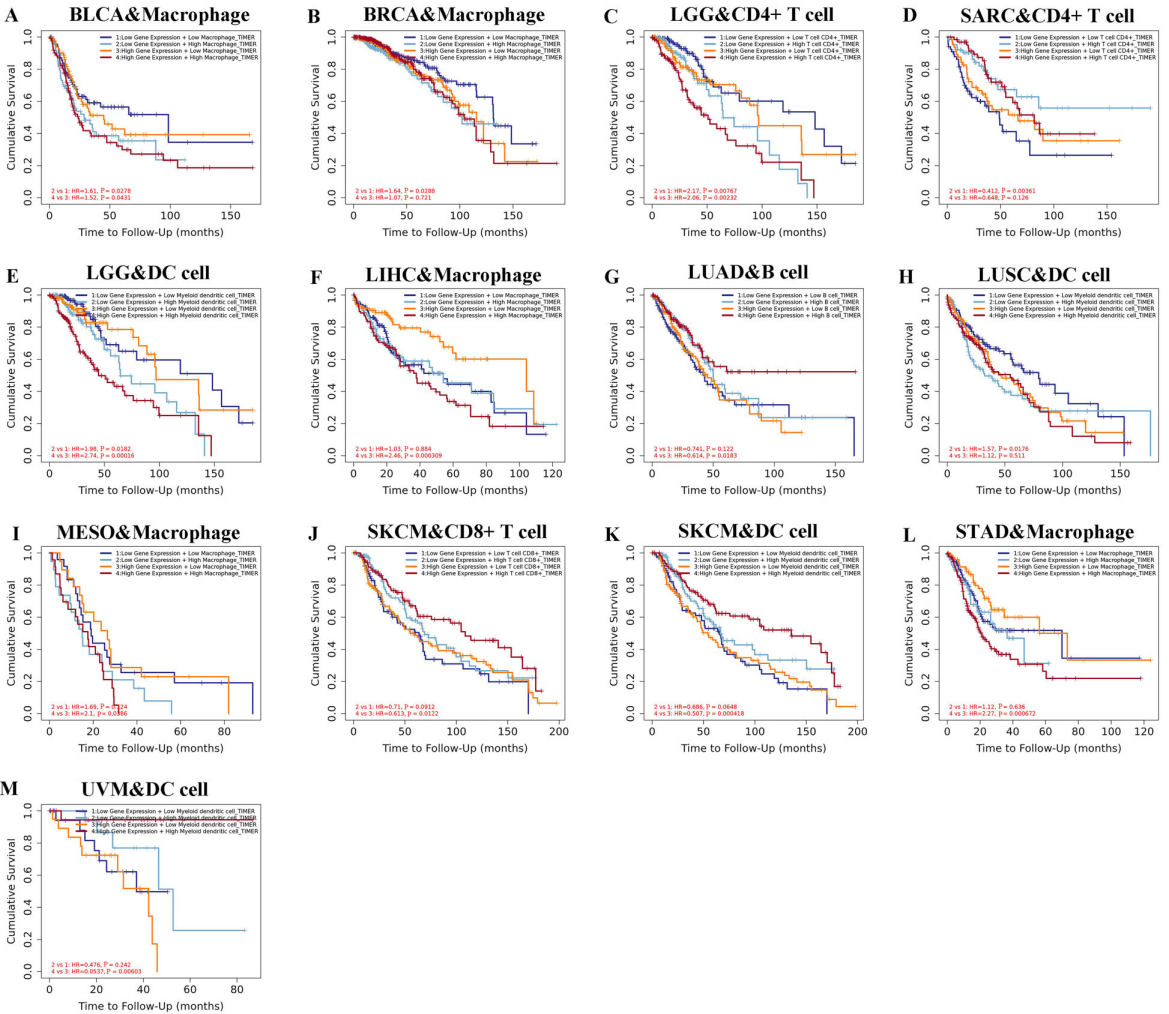
The relationship between FOXO1 expression combined with immune cells score and overall survival (OS) in pan-cancer. See Table 1 for abbreviations. **A**, OS analysis of FOXO1 expression combined with macrophage score in BLCA and **B**, BRCA. **C**, OS analysis of FOXO1 expression combined with T cell CD4+ score in LGG and **D**, SARC. **E**, OS analysis of FOXO1 expression combined with myeloid dendritic cell score in LGG. **F**, OS analysis of FOXO1 expression combined with macrophage score in LIHC. **G**, OS analysis of FOXO1 expression combined with B cell score in LUAD. **H**, OS analysis of FOXO1 expression combined with myeloid dendritic cell score in LUSC. **I**, OS analysis of FOXO1 expression combined with macrophage score in MESO. **J**, OS analysis of FOXO1 expression combined with T cell CD8+ score in SKCM. **K**, OS analysis of FOXO1 expression combined with myeloid dendritic cell score in SKCM. **L**, OS analysis of FOXO1 expression combined with macrophage score in STAD. **M**, OS analysis of FOXO1 expression combined with myeloid dendritic cell score in UVM.

Additionally, the association between FOXO1 expression, immune cell infiltration, and OS was investigated using Kaplan-Meier analysis. Lower scores of macrophage infiltration in BLCA were associated with improved overall survival, regardless of the expression level of FOXO1 (as shown in Figure 9A). Lower infiltration scores of macrophages in BRCA were associated with improved overall survival, regardless of the expression level of FOXO1 (Figure 9B). Lower infiltration scores of CD4+ T cell in LGG were associated with improved overall survival, regardless of the FOXO1 expression level (Figure 9C). Patients with low FOXO1 expression exhibited improved OS when they had increased infiltration scores of CD4+ T cell in LGG. In patients with elevated FOXO1 levels, there was no notable association between CD4+ T cell infiltration score and OS (Figure 9D). Lower myeloid dendritic cell infiltration scores in LGG were associated with improved overall survival, regardless of the FOXO1 expression level (Figure 9E). In patients with high FOXO1 expression, lower macrophage infiltration scores were associated with improved OS for LIHC. In the case of patients exhibiting low FOXO1 levels, there was no notable association between macrophage infiltration score and OS (Figure 9F). In patients with high FOXO1 expression, better OS was observed in LUAD cases with higher scores of B cell infiltration. In patients with low FOXO1 expression, there was no significant correlation between the B cell infiltration score and OS (Figure 9G). In patients with low FOXO1 expression, better OS was observed for LUSC with lower myeloid dendritic cell infiltration scores. In patients with elevated FOXO1 levels, there was no notable association between myeloid dendritic cell infiltration score and OS (Figure 9H). In patients with high FOXO1 expression, better overall survival was observed with lower macrophage infiltration scores in MESO. In the case of patients exhibiting low FOXO1 levels, there was no notable association between macrophage infiltration score and OS (Figure 9I). In SKCM, improved overall survival was observed with increased infiltration of CD8+ T cells, regardless of the FOXO1 expression level (Figure 9J). In SKCM, improved overall survival was observed with higher myeloid dendritic cell infiltration scores, regardless of the expression level of FOXO1 (Figure 9K). In patients with high FOXO1 expression, a higher OS was observed when there were lower macrophage infiltration scores in STAD. In the case of patients exhibiting low FOXO1 levels, there was no notable association between macrophage infiltration score and OS (Figure 9L). In patients with high FOXO1 expression, higher myeloid dendritic cell infiltration scores were associated with improved OS for UVM. In patients exhibiting low FOXO1 levels, there was no notable association between myeloid dendritic cell infiltration score and OS (Figure 9M).

### Gene enrichment analysis related to FOXO1 function

**Figure 10:**
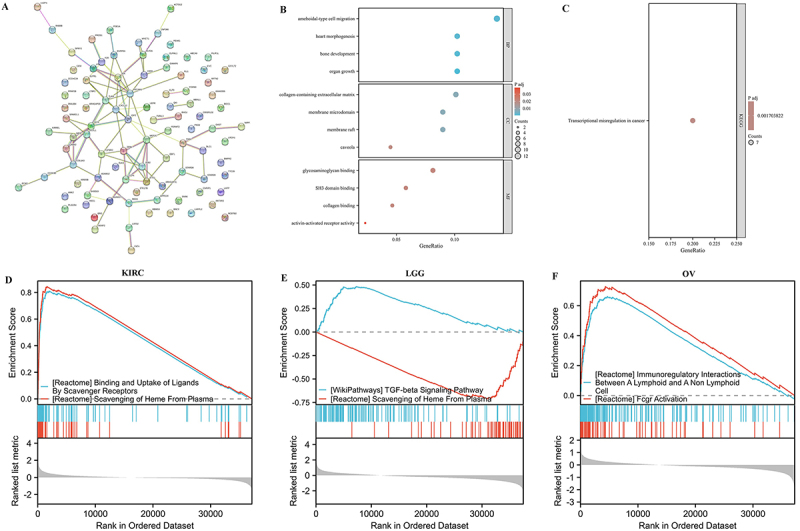
Functional enrichment analysis of FOXO1-related genes. **A**, Co-representation network map according to the Top 100 FOXO1-related genes. **B**, GO enrichment analysis according to the Top 100 FOXO1-related genes. **C**, KEGG pathways analysis according to the Top 100 FOXO1-related genes. **D**-**F**, GSEA according to the differentially expression analysis in kidney renal clear cell carcinoma (KIRC), low grade glioma (LGG), and ovarian carcinoma (OV).

In order to understand how FOXO1 could biologically contribute to various cancers, the PPI network was acquired from the STRING database based on the top 100 genes associated with FOXO1 (Figure 10A). The GO enrichment analysis indicated that genes associated with FOXO1 may have functions in the biological processes of ‘ameboidal-type cell', ‘heart morphogenesis', ‘bone development', and ‘origin growth'. These processes are involved in ‘collagen-containing extracellular matrix', ‘membrane microdomain', ‘membrane raft', ‘caveola', ‘glycosaminoglycan binding', ‘SH3 domain binding', ‘collagen binding', and ‘activin-activated receptor activity' (Figure 10B). According to the KEGG pathway analysis, genes associated with FOXO1 may have functions in the development of ‘transcriptional misregulation in cancer' (Figure 10C).

### Gene set enrichment analysis

To further clarify the potential biological role of FOXO1 in these tumors, the GSEA was performed in LGG, KIRC, and OV. In KIRC, FOXO1 was associated with the ‘attachment and absorption of molecules by scavenger receptors' and ‘removal of heme from plasma' (Figure 10D). In LGG, FOXO1 was associated with the ‘TGF-beta signaling pathway' and the ‘removal of heme from plasma' (Figure 10E). In OV, FOXO1 was associated with the ‘immunomodulatory interactions between a lymphoid and a non-lymphoid cell' and ‘activation of Fcgr' (Figure 10F).

## Discussion

Adipose tissues primarily exhibit FOXO1 expression (12). FOXO1 possesses a DNA-binding domain with a fork-like structure (FHD), a sequence for exporting from the nucleus (NES), a signal for localizing the nucleus (NLS), and a domain for activating transcription (TAD) (13). It has been reported that FOXO1 could affect the apoptosis, proliferation, invasion, and migration of tumors. The investigation of FOXO1 expression and functions in pan-cancer has not been conducted before.

In this research, we examined the expression of FOXO1 in pan-cancer using the TCGA databases. In some tumors, the expression of FOXO1 was significantly lower in tumor tissue compared with normal tissues, both in unpaired and paired samples. These tumors included BLCA, BRCA, COAD, KIRC, KIRP, LIHC, LUAD, LUSC, PRAD, READ, THCA, and UCEC. Figure 2 shows that the protein expression of FOXO1 was higher in normal tissues in LUAD, STAD, LIHC, PAAD, KIRC, and OV. The result of mRNA expression level of FOXO1 came from the TCGA database. The images of Figure 2 came from the HPA database. These images show that the expression of FOXO1 was higher in normal tissues compared with the tumor tissue in these cancers. The above results came from different databases, which indicated that both the mRNA expression level of FOXO1 and the protein expression level of FOXO1 were higher in normal tissues compared with tumor tissues in pan-cancer.

Additionally, the TCGA database was used to perform prognostic analysis for FOXO1 across various cancer types. The findings indicated that increased FOXO1 expression in cancerous tissues was associated with worse OS in LGG and OV, but with improved OS in KIRC. In contrast, OS was not correlated with the expression level of FOXO1 in tumor tissues in other types of tumors. According to reports, reduced FOXO1 levels in cancerous tissues were found to be strongly associated with metastasis and poorer survival rates in KIRC (14). This finding is in line with our OS analysis, which indicated that FOXO1 might be a cancer suppressor gene in KIRC. In LGG, there was no relevant literature. But the OS analysis result was consistent with the result from the TCGA database that the expression of FOXO1 was significantly increased in tumor tissues, which indicated that FOXO1 might play a role of oncogenic gene in LGG. For OV, it has been reported that FOXO1 was ubiquitously expressed in different OV cell lines and knockdown of FOXO1 could inhibit the proliferation of these OV cell lines (15). This finding was consistent with our OS analysis, which indicated that FOXO1 might also play the role of an oncogenic gene in OV. Expanding the quantity was required to confirm the expression of FOXO1 in OV. In LGG, OV, and KIRC, the analysis of DSS and PFI aligned with the findings of OS analysis. Additionally, we observed a correlation between the expression of FOXO1 in tumor tissues and DSS or PFI in other types of tumors.

Currently, an increasing amount of research has indicated that the infiltration of immune cells plays a role in the prognosis of tumors (16). To explore the possible impact of FOXO1 on tumor prognosis, the correlation between infiltration of immune cells and FOXO1 expression was examined in LGG, KIRC, and OV. In LGG, the expression of FOXO1 showed a strong association with the presence of myeloid dendritic cells and macrophages, individually. In addition, the analysis of operating systems revealed that LGG had improved OS when there were lower scores for infiltration of myeloid dendritic cells, regardless of the expression level of FOXO1. The findings suggested that FOXO1 could potentially impact the OS of LGG by affecting the presence of myeloid dendritic cells. However, the macrophage expression level did not have a significant impact on the overall survival in LGG. In KIRC and OV, we observed significant associations between FOXO1 expression and the infiltration of certain immune cells. However, the analysis of OS revealed no notable disparities in these tumors when considering the varying levels of immune cell expression. Additional investigation is required to uncover the possible mechanisms.

Functional enrichment analysis and gene set enrichment analysis were performed to elucidate the potential biological role of FOXO1 in pan-cancer. According to the analysis of the KEGG pathway, genes associated with FOXO1 could potentially have functions in transcription disorder in cancer, whereas FOXO1 itself solely functions as a regulator of transcription. FOXO1 was also associated with the ‘immunoregulatory interactions involving a lymphoid and a non-lymphoid cell' and the ‘TGF-beta signaling pathway', suggesting potential involvement of FOXO1 in tumor development.

This is the first study to research the expression and mechanism of FOXO1 in pan-cancer. The prognosis of multiple tumors, particularly LGG, OV, and KIRC, was found to be linked with FOXO1. FOXO1 might play a role as an oncogenic gene in LGG and OV, while playing a cancer suppressor role in KIRC. The establishment of nomogram models has proven to be highly accurate in predicting OS in patients with LGG, OV, and KIRC. The infiltration of certain immune cells in LGG, OV, and KIRC showed significant correlations with the expression of FOXO1. Through the analysis of OS by combining FOXO1 expression and the infiltration of immune cells, we discovered that the presence of FOXO1 could potentially impact the overall survival of LGG through the infiltration of either myeloid dendritic cells or CD4+ T cells. In other tumors, FOXO1 might also affect the OS through the infiltration of some immune cells. Analysis of functional enrichment and gene sets indicated that FOXO1 may play roles in the development of tumors through interactions between immune and non-lymphoid cells, the TGF-beta signaling pathway, and the misregulation of transcription in cancer.

Of course, there are limitations to this study. First, our research was based on public databases. The quality of data collection could be inconsistent in different databases, which might affect the results of some analyses. Secondly, our study was based on bioinformatics, and the results had not been verified experimentally or clinically. Further investigations are required to elucidate the function and mechanisms of FOXO1 in all types of cancer.

### Conclusion

In brief, our findings indicated that FOXO1 is linked to the prognosis of various tumors, particularly in LGG, OV, and KIRC. FOXO1 may exert its function in these tumors by controlling the immune microenvironment. Additional investigations revealed that FOXO1 could impact the onset and progression of tumors via interactions that regulate the immune system between a cell of the lymphatic system and a cell outside the lymphatic system, the TGF-beta signaling pathway, and the misregulation of gene transcription in cancer. Nevertheless, further investigations are required to elucidate the function and mechanism of FOXO1 in all types of cancer.
